# Supplementary material for: Bootstrapping quantum process tomography via a perturbative ansatz
Source: Nat Commun. 2020 Feb 27;11:1084. doi: 10.1038/s41467-020-14873-1 (PMC7046656; doi:10.1038/s41467-020-14873-1)
Supplement: Supplementary file 1 — Supplementary Information [file 41467_2020_14873_MOESM1_ESM.pdf]

Supplementary Information for: Bootstrapping quantum process tomography via a perturbative ansatz

L. C. G. Govia et al.

Supplementary Note I: Device Parameters

Supplementary Table I shows device performance for all five qubits on the sample. Note for the experiment presented only Q_1, Q_2 and Q_3 were used. All gate times are 80 ns.

Qubit	f_{01} (GHz)	T_2^* (μs)	T_1 (μs)	ZZ (kHz)
Q_1	5.3067	21 ± 5	43 ± 9	30 (Z_1Z_4); 25 (Z_1Z_2)
Q_2	5.2125	11 ± 3	8.3 ± 0.3	8 (Z_2Z_3)
Q_3	5.357	43 ± 9	50 ± 6	35 (Z_3Z_5)
Q_4	5.4177	20 ± 7	44 ± 6	32 (Z_2Z_4)
Q_5	5.4123	22 ± 6	45 ± 3	72 (Z_2Z_5)

Supplementary Table I. Qubit frequencies and coherence times. Q_4 and Q_5 were not used in this experiment. Coherence times are quoted as average and standard deviation of the values measured over a $\sim$ 16 h span. A window of 2 h was not included in the statistics for Q_1, during which T_1 was suppressed most likely due to a TLS moving into resonance with the qubit [1]. Static ZZ interaction strengths are reported for qubit pairs that are coupled through a common bus resonator.

Supplementary Note II: Pseudocode for PAPA

Algorithm 1 PAPA reconstruction of an N qubit quantum process $\mathcal{E}$

```
Pairwise process tomography:
for j < N do
    for k < j do
        S = {j, k}
        Prepare spectator qubits in I/2
        σ_S = Two-qubit QPT of E for pair S
        k += 1
    end for
    j += 1
end for

Boostrapping reconstruction:
define χ⃗ = χ-matrix PAPA free parameters
define [cost(χ⃗)]_nm = |[σ_S]_nm − [ρ_S(χ⃗)]_nm|^2
define constraints(χ⃗) = CPTP constraints
χ⃗^est = arg min cost(χ⃗) + constraints(χ⃗)
return χ⃗^est
```

See Supplementary Note VI for details on the constraints used in the minimization.

Supplementary Note III: PAPA+GST N-qubit Gates

In this appendix we discuss the set of N-qubit gates that can be characterized via bootstrapping with PAPA+GST. We will focus on the $N = 3$ case since the extension to $N > 3$ is straightforward from the three-qubit results. Consider an ideal three-qubit gate written as

$$\hat{U} = \sum_{ijk} u_{ijk} \hat{U}_i \otimes \hat{U}_j \otimes \hat{U}_k, \tag{1}$$

where $\mathrm{Tr}(\hat{U}_i \hat{U}_j^\dagger) = 2\delta_{ij}$ such that $\{\hat{U}_i\}$ is an orthonormal basis for one-qubit operator space. We will label the ideal process for this gate as $\mathcal{U}$, and label the imperfect experimental implementation of this process as $\tilde{\mathcal{U}}$. For notational simplicity we break slightly from the nomenclature used in the main text, and throughout this appendix processes without tildes will be ideal, and those with tildes will be experimental implementations of the ideal process.

The Choi state of the ideal process is

$$\rho_{\mathcal{U}} = \frac{1}{8} \sum_{\mu\nu} |\psi_\mu\rangle\langle\psi_\nu| \otimes \sum_{\substack{ijk \\ i'j'k'}} u_{ijk} u_{i'j'k'} \hat{U}_i \otimes \hat{U}_j \otimes \hat{U}_k \, |\psi_\mu\rangle\langle\psi_\nu| \, \hat{U}_{i'}^\dagger \otimes \hat{U}_{j'}^\dagger \otimes \hat{U}_{k'}^\dagger. \tag{2}$$

Then, as an example, the two-qubit reduction for qubits 1-2 is given by

$$\begin{aligned} \rho_{\mathcal{U}_{1,2}} &= \frac{1}{4} \sum_{\mu\nu} |\psi_\mu\rangle\langle\psi_\nu| \otimes \sum_{\substack{ij \\ i'j'}} \sum_{k} u_{ijk} u_{i'j'k} \hat{U}_i \otimes \hat{U}_j \, |\psi_\mu\rangle\langle\psi_\nu| \, \hat{U}_{i'}^\dagger \otimes \hat{U}_{j'}^\dagger \\ &= \frac{1}{4} \sum_{\mu\nu} |\psi_\mu\rangle\langle\psi_\nu| \otimes \mathcal{U}_{12}(|\psi_\mu\rangle\langle\psi_\nu|), \end{aligned} \tag{3}$$

where we have used the fact that $\mathrm{Tr}(\hat{U}_i \hat{\mathbb{I}} \hat{U}_j^\dagger) = 2\delta_{ij}$, and $\mathcal{U}_{12}$ is the two-qubit process defined by

$$\mathcal{U}_{12}(\rho) = \sum_{k} u_{ijk} u_{i'j'k} \hat{U}_i \otimes \hat{U}_j \rho \hat{U}_{i'}^\dagger \otimes \hat{U}_{j'}^\dagger. \tag{4}$$

The general PAPA approach would be to characterize the experimental implementation of the process $\mathcal{U}_{12}$, i.e. $\tilde{\mathcal{U}}_{12}$, via two-qubit QPT on qubits 1-2 when $\tilde{\mathcal{U}}$ occurs. The PAPA+GST approach is the situation where one *does not* want to perform two-qubit QPT for every unknown three-qubit process, but would rather bootstrap characterizations of three-qubit processes from existing two-qubit gate set characterizations.

In the PAPA+GST approach, the two-qubit reduction $\rho_{\tilde{\mathcal{U}}_{1,2}}$ can be experimentally characterized if the ideal process $\mathcal{U}_{12}$ can be described as a convex sum of unitary

processes

$$\mathcal{U}_{12}(\rho) = \sum_i c_i \hat{G}_i \rho \hat{G}_i^\dagger, \tag{5}$$

with each $\hat{G}_i$ in the GST characterized gate set. If this is the case, then

$$\rho_{\tilde{\mathcal{U}}_{1,2}} = \sum_i c_i \sigma_{\tilde{\mathcal{G}}_i}, \tag{6}$$

where $\tilde{\mathcal{G}}_i$ is the experimental implementation of the gate $\hat{G}_i$, and each $\sigma_{\tilde{\mathcal{G}}_i}$ can be obtained from the GST gate set which contains all $\tilde{\mathcal{G}}_i$.

For the ideal gate set we have used in the main text, CNOT + $\{\hat{\mathbb{I}}, \hat{X}, \hat{Y}, \hat{Z}\}^{\otimes 2}$, we will now show that any three-qubit quantum gate consisting of a single-layer circuit of these gates can be characterized using PAPA+GST. Three-qubit gates of the form $\hat{G}_1 \otimes \hat{G}_2 \otimes \hat{G}_3 \in \{\hat{\mathbb{I}}, \hat{X}, \hat{Y}, \hat{Z}\}^{\otimes 3}$ can obviously be parameterized by PAPA, as one can trivially show that the two-qubit processes to be characterized are the unitary gates $\hat{G}_1 \otimes \hat{G}_2$, $\hat{G}_1 \otimes \hat{G}_3$, and $\hat{G}_2 \otimes \hat{G}_3$, which are all in the GST gate sets.

For a three-qubit gate that involves a CNOT on two of the qubits, a bit more effort is required to show that the necessary two-qubit gates to be characterized are still in the GST gate set. For example, consider the ideal gate $\hat{U} = \mathrm{CNOT}_{12} \otimes \hat{\mathbb{I}}$ used in the main text. This has the ideal two-qubit reduced dual states

$$\rho_{\mathcal{U}_{1,2}} = \frac{1}{4} \sum_{\mu\nu} |\psi_\mu\rangle\langle\psi_\nu| \otimes \mathrm{CNOT}\, |\psi_\mu\rangle\langle\psi_\nu|\, \mathrm{CNOT} \tag{7}$$

$$\rho_{\mathcal{U}_{1,3}} = \frac{1}{2} \left(\rho_{\mathbb{I}\otimes\mathbb{I}} + \rho_{Z\otimes\mathbb{I}}\right) \tag{8}$$

$$\rho_{\mathcal{U}_{2,3}} = \frac{1}{2} \left(\rho_{\mathbb{I}\otimes\mathbb{I}} + \rho_{X\otimes\mathbb{I}}\right) \tag{9}$$

and therefore the necessary gates to characterize are CNOT for qubits 1-2, $\hat{\mathbb{I}} \otimes \hat{\mathbb{I}}$ and $\hat{Z} \otimes \hat{\mathbb{I}}$ for qubits 1-3, $\hat{\mathbb{I}} \otimes \hat{\mathbb{I}}$ and $\hat{X} \otimes \hat{\mathbb{I}}$ for qubits 2-3. As all of these gates belong to their respective GST gate sets, a characterization of $\hat{U}$ can be bootstrapped using PAPA+GST. It is straightforward to show that this generalizes to all arrangements of the CNOT (i.e. on any pair of qubits), and any gate on the qubit not involved in the CNOT.

So far we have only commented on the ideal two-qubit gates that need to be characterized for a given N-qubit process, and not on the other criteria for PAPA+GST, namely tolerable error. The general criteria is not as strong as all error needing to be gate-independent. For instance, three-qubit gates such as $\hat{G}_1 \otimes \hat{G}_2 \otimes \hat{G}_3$ may have error that is dependent on the specific single-qubit gates implemented, as this will be captured in characterization of the two-qubit reductions. Similarly, if the error in a single-qubit gate depends on the gate implemented on another qubit (i.e. context dependence) this will also be captured by PAPA+GST.

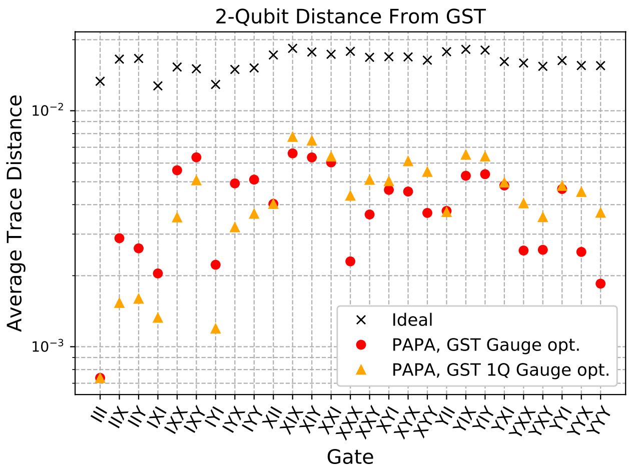

Supplementary Figure 1. Comparison of the GST measured process matrices to the ideal process matrices, and to the PAPA reconstructions with or without additional gauge optimization to bring the pairwise GST gate sets into consistent single-qubit gauges. Red circles are the data points from the main text with GST gauge optimization, and orange triangles are the PAPA reconstruction with the single-qubit gauge optimization. PAPA data points are the average trace distance of the three reduced processes from there corresponding GST characterizations.

The fact that both gate-dependent and context-dependent error fits within the PAPA+GST framework for simultaneous single-qubit gates comes from the fact that the physical implementation of the simultaneous single-qubit gates on N qubits is the same as on two-qubits. This is often not the case for an entangling gate such as $\mathrm{CNOT}_{12} \otimes \hat{\mathbb{I}}$, where the physical implementation, such as a CR-CNOT, could be vastly different than the physical implementation of the gates in its reduced two-qubit decomposition, cf. Supplementary Eqs. (7)-(9).

This is especially true in the case of the CR-CNOT where the error model assumed – over-rotation plus cross-talk – is intrinsic to the CR interaction. As such, GST characterization of the simultaneous single-qubit gates $\hat{Z} \otimes \hat{\mathbb{I}}$ and $\hat{X} \otimes \hat{\mathbb{I}}$ (on qubit pairs 1-3 and 2-3 respectively) would not contain any signature of this error. This makes PAPA+GST impossible, as two qubit tomography from GST would be inconsistent across qubit pairs. Even a large difference in gate-length between entangling and single-qubit gates can result in an error discrepancy due to decoherence, and this is enough to make PAPA+GST inapplicable. In such situations standard PAPA should be used in combination with other SPAM-insensitive two-qubit QPT techniques.

Supplementary Note IV: Single Qubit Gauge Optimization

It is not possible to gauge optimize the pairwise GST characterized gate sets to one another, since they are two-qubit gate sets that are on different pairs of qubits, with at most one qubit shared between gate sets. With that in mind, what can be done is to ensure that gates shared by different pairwise gate sets are gauge consistent. For the gate set we considered in our experiment, the shared gates are those with one non-identity gate. For example, in a three-qubit system, the gate $\hat{X}_1 \otimes \hat{\mathbb{I}}_2$ in gate set 1-2 is the same as the gate $\hat{X}_1 \otimes \hat{\mathbb{I}}_3$ in the gate set 1-3.

For every pairwise gate set we create two single qubit gate sets by calculating the reduced single-qubit gates from two-qubit gates of the form $\mathcal{G}_{G_k,\mathbb{I}_n}$ and $\mathcal{G}_{\mathbb{I}_k,G_n}$, which results in $(N^2 - N)$ single-qubit gate sets total. For each qubit we choose one of these gate sets to be the "true gauge", and gauge optimize the other single-qubit gate sets to this one. This requires $(N^2 - 2N)$ total gauge optimizations.

Using the calculated gauge transformations, we bring the GST gate sets into a consistent single-qubit gauge. For example, in our three qubit system, we take gate set 1-3 to be in the true gauge for qubits 1 and 3 and gate set 2-3 to be in the true gauge for qubit 2. We therefore define the single-qubit gauge consistent gate sets

$$\mathrm{GS}'_{13} = \mathrm{GS}_{13} \tag{10}$$

$$\mathrm{GS}'_{12} = \hat{T}_1^{-1} \otimes \hat{T}_2^{-1} \mathrm{GS}_{12} \hat{T}_1 \otimes \hat{T}_2 \tag{11}$$

$$\mathrm{GS}'_{23} = \hat{\mathbb{I}} \otimes \hat{T}_3^{-1} \mathrm{GS}_{23} \hat{\mathbb{I}} \otimes \hat{T}_3 \tag{12}$$

where $\hat{T}_k$ is the gauge transformation for qubit k. We use these new gate sets in a PAPA reconstruction, the results of which are shown in Supplementary Fig. 1. As can be seen, the single-qubit gauge optimization improves the quality of the reconstruction for three-qubit gates with only one non-identity gate, but does worse for gates with multiple non-identity components.

Supplementary Note V: Further Numerical Simulations

To further test our PAPA approach for multi-qubit QPT, we numerically simulate "unknown" three-qubit processes, and then reconstruct the PAPA characterization of these processes. We consider several example processes formed by one of the ideal three-qubit gates $\hat{\mathbb{I}} \otimes \hat{\mathbb{I}} \otimes \hat{\mathbb{I}}$, $\mathrm{CNOT}_{12} \otimes \hat{\mathbb{I}}$, or $\hat{X} \otimes \hat{Y} \otimes \hat{X}$, followed by either a coherent or incoherent error process.

In standard PAPA reconstruction, pairwise two-qubit QPT is used to characterize the reduced two-qubit process, and obtain $\sigma_{\mathcal{S}}$ for each qubit pair. With PAPA+GST this is circumvented by using a GST characterized gate set for each qubit pair to calculate $\sigma_{\mathcal{S}}$, provided the ideal reduced two-qubit process can be built from gates in the ideal gate set. For the example three-qubit ideal gates chosen, the required two-qubit gates are contained in the ideal gate set CNOT $+ \{\hat{\mathbb{I}}, \hat{X}, \hat{Y}, \hat{Z}\}^{\otimes 2}$. We follow the PAPA+GST approach for these numerical tests, simulating the implementation of this gate set on all qubit pairs, including the error process, and use results of these simulations as our GST reconstructed two-qubit gate sets. We then use the characterized two-qubit gate sets to calculate $\sigma_{\mathcal{S}}$ for each qubit pair. In Supplementary Note III we discuss the three-qubit gate decompositions describing the two-qubit gate sets to be characterized.

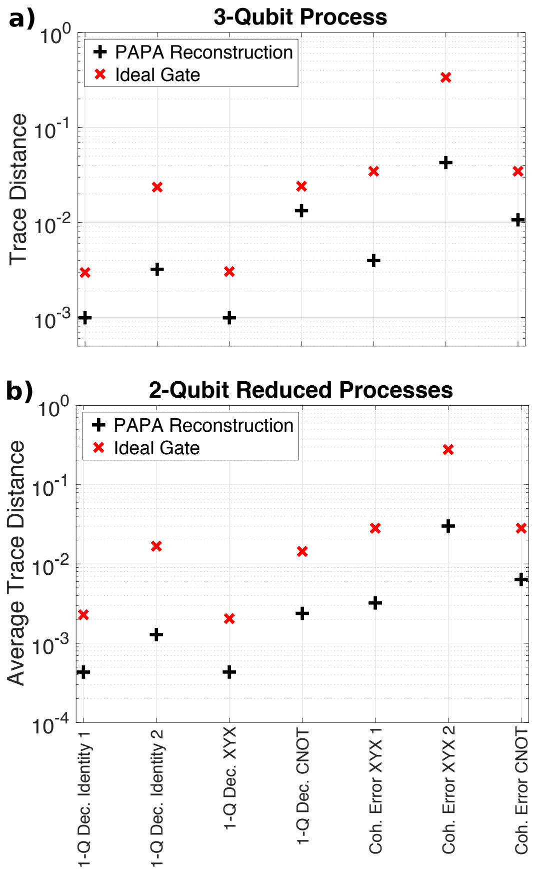

Supplementary Figure 2. **a)** Simulated trace distance between the actual three-qubit process Choi state and either the PAPA reconstructed Choi state (black +) or the ideal gate (red ×), see Supplementary Eq. (16). **b)** The average trace distance between the reduced two-qubit Choi states, see Supplementary Eq. (17). Processes i), ii) are the all identity gate of length 50 ns and 400 ns; iii), v), and vi) are $\hat{X} \otimes \hat{Y} \otimes \hat{X}$ of length 50 ns; iv) and vii) are $\mathrm{CNOT}_{12} \otimes \hat{\mathbb{I}}$ of length 400 ns. i), ii), iii), and iv) have single-qubit decoherence with $T_1 = T_2 = 50$ μs; v) and vii) have coherent error $\phi = 0.02$ and vi) has $\phi = 0.2$.

For the results shown in Supplementary Fig. 2 we consider two cases of gate-independent error processes, either a coherent error described by single-qubit rotations on all

three qubits

$$\hat{G}_{\text{Coh. Error}} = \hat{X}_\phi \otimes \hat{Y}_\phi \otimes \hat{X}_\phi \tag{13}$$
$$\hat{X}_\phi = \cos(\phi)\hat{\mathbb{I}} + i\sin(\phi)\hat{X} \tag{14}$$
$$\hat{Y}_\phi = \cos(\phi)\hat{\mathbb{I}} + i\sin(\phi)\hat{Y} \tag{15}$$

or single-qubit decay and pure dephasing implemented by their standard Kraus operator representations [2].

We compare the PAPA+GST reconstruction for a noisy gate to the actual simulated noisy gate by calculating the trace distance between the Choi state of the PAPA-reconstructed three-qubit process, $\rho_{\mathcal{E}}$, and that for the actual process, $\rho_{\mathcal{E}}^{\text{act}}$

$$\text{Trace Dist.} = \frac{1}{2}\text{Tr}\left[\sqrt{\left(\rho_{\mathcal{E}} - \rho_{\mathcal{E}}^{\text{act}}\right)^\dagger\left(\rho_{\mathcal{E}} - \rho_{\mathcal{E}}^{\text{act}}\right)}\right]. \tag{16}$$

In experiment we do not have access to the actual full three-qubit process, and cannot use the N-qubit trace distance as a performance metric. Instead, in the main text we compared each of the reduced two-qubit processes from PAPA, to the actual two-qubit reduced processes from GST, and we do the same for our numerical tests, via the expression used in the main text

$$\text{Trace Dist.} = \frac{1}{2}\text{Tr}\left[\sqrt{\left(\rho_{\mathcal{S}} - \sigma_{\mathcal{S}}\right)^\dagger\left(\rho_{\mathcal{S}} - \sigma_{\mathcal{S}}\right)}\right]. \tag{17}$$

The results of both trace distance calculations are shown in Supplementary Fig. 2 for the seven candidate processes listed in the caption.

As the results show, the PAPA reconstructed process always improves upon the initial guess (ideal gate), both in terms of the trace distance for the full three-qubit process reconstruction, Supplementary Fig. 2a), and the average of the trace distances for the two-qubit reconstructions, Supplementary Fig. 2b). This improvement is typically around one order of magnitude, except in the case of the CNOT gate, which was the most difficult to reconstruct of the gates tested. The PAPA reconstructions for these numerical simulations were done using the `MATLAB` implementation.

Supplementary Note VI: Numerical Implementation

The computational task in PAPA characterization is the simultaneous solution of $\rho_{\mathcal{S}} = \sigma_{\mathcal{S}}$ for each pairwise reduction, from which we obtain the elements of the two-qubit χ-matrices, $\chi_{i_{k,n}}^{j_{k,n}}$. These equations are nonlinear in general, and must be solved under the constraint that each of the two-qubit χ-matrices describes a completely-positive and trace-persevering (CPTP) map.

This implies that the χ-matrix is a positive semi-definite matrix with trace 4 (dimension of two-qubit

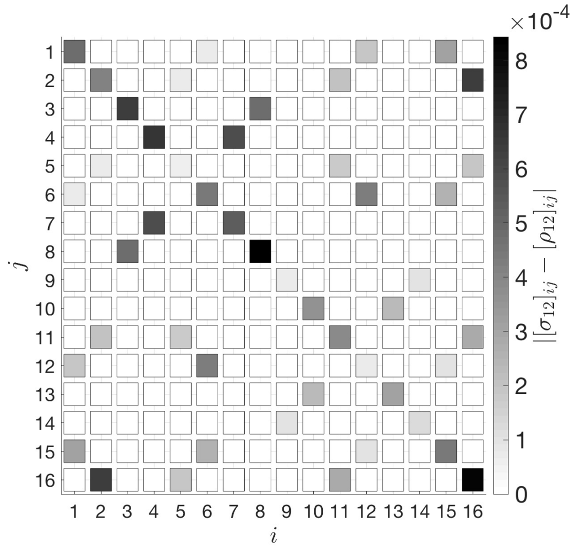

Supplementary Figure 3. Absolute value of the element-wise difference between the "measured" Choi state, σ_{12}, and the PAPA reconstructed Choi state, ρ_{12}, for the effective process experienced by qubit pair 1-2 during a CNOT gate with single-qubit decoherence. Simulation parameters for the three-qubit process are the same as in the main text.

Hilbert space). Further, TP requires an additional constraint, which to describe we need to parameterize a two-qubit process on the set $\mathcal{S}$ in the usual way via its χ-matrix

$$\mathcal{E}_{\mathcal{S}}(\rho) = \sum_{p,r}^{16}[\boldsymbol{\chi}_{\mathcal{S}}]_{p,r}\hat{E}_r\rho\hat{E}_p^\dagger, \tag{18}$$

where $\{\hat{E}_p\}$ is a basis for two-qubit operator space. The TP constraint is then [2]

$$\sum_{p,r}^{16}[\boldsymbol{\chi}_{\mathcal{S}}]_{p,r}\hat{E}_p^\dagger\hat{E}_r - \hat{\mathbb{I}}\otimes\hat{\mathbb{I}} = 0. \tag{19}$$

Comparing this to our previous parameterization of a two-qubit process in terms of one-qubit processes used in the main text,

$$\mathcal{E}_{\mathcal{S}} = \sum_{i,j_{k,n}}^{16}\chi_{i_{k,n}}^{j_{k,n}}\mathcal{A}_{i_{k,n}}^{(k)}\otimes\mathcal{A}_{j_{k,n}}^{(k+n)}, \tag{20}$$

for $\mathcal{S} = \{k, k+n\}$, we see that the two parameterizations are related by splitting each index $i_{k,n}$ and $j_{k,n}$ into two parts via the equations

$$\begin{aligned}
&\mathcal{A}_{i_{k,n}}^{(k)}\otimes\mathcal{A}_{j_{k,n}}^{(k+n)}(\rho) = \hat{E}_{r_1}\otimes\hat{E}_{r_2}\rho\hat{E}_{p_1}^\dagger\otimes\hat{E}_{p_2}^\dagger = \hat{E}_r\rho\hat{E}_p^\dagger,\\
&i_{k,n}\to(i,i') \quad j_{k,n}\to j,j',\\
&r = (r_1,r_2) = (i,j) \quad p = (p_1,p_2) = (i',j'),\\
&[\boldsymbol{\chi}_{\mathcal{S}}]_{p,r} = \chi_{(r_1,p_1)}^{(r_2,p_2)}.
\end{aligned}$$

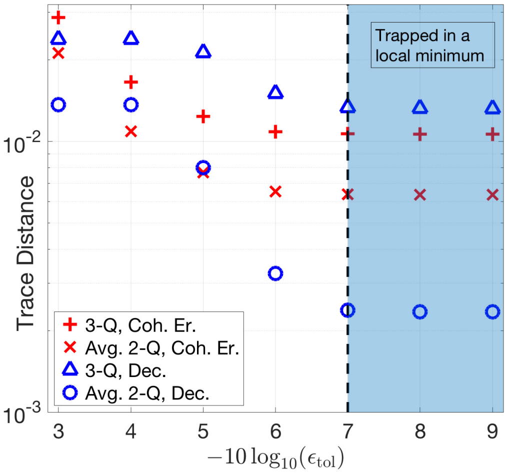

Supplementary Figure 4. Trace distance between the simulated Choi state and PAPA reconstructed Choi state for the three-qubit process, and reduced two-qubit processes as a function of the solver tolerance, $\epsilon_{\rm tol}$. The CNOT gate with coherent error (red $+$ and $\times$) and decoherence (blue triangles and circles) were used for these simulations. The vertical dashed line indicates the tolerance used for the simulations presented in the main text.

To solve for the χ-matrix elements under the CPTP constraints, we use a least-squares minimization approach implemented in MATLAB [3]. Here, the cost function for the least-squares minimization consists of two parts. The first encodes the experimental characterizations of the two-qubit reductions, and simply consists of the element-wise difference between the two-qubit reduced Choi state for each pair of qubits and the current estimate for the two-qubit reduced Choi state generated by PAPA,

$$C_1\left[\vec{\chi}\right] = \sum_{\mathcal{S}}\sum_{k,n}\left|\left[\rho_{\mathcal{S}}(\vec{\chi})\right]_{k,n} - \left[\sigma_{\mathcal{S}}\right]_{k,n}\right|^2, \tag{21}$$

where $\vec{\chi}$ is a vector of the χ-matrices for the processes on all qubit pairs that make up the PAPA, and the sum over $\mathcal{S}$ runs over all qubit pairs.

The second part of the cost function, $C_2\left[\vec{\chi}\right]$, encodes the CPTP constraints, and consists of the difference between the trace of each χ-matrix estimate and the Hilbert space dimension (in this case 4), the sum of all negative eigenvalues of the χ-matrix estimate (to constrain positivity), and the elements of Supplementary Eq. (19). The least-squares minimization solves the problem

$$\vec{\chi}^{\rm est} = \arg\min_{\vec{\chi}}\left(C_1\left[\vec{\chi}\right] + C_2\left[\vec{\chi}\right]\right). \tag{22}$$

Even with the CPTP constraints imposed, the χ-matrix estimates returned by the numerical solver will not necessarily be positive semi-definite. As such, we apply a post-processing step where we diagonalize each χ-matrix estimate, generating a set of eigenvalues λ_i with corresponding eigenvectors $|v_i\rangle$. We can then create a positive semi-definite χ-matrix estimate for each two-qubit process

$$\tilde{\chi}_{\mathcal{S}}^{\rm est} = \sum_{\lambda_i\geq 0}\lambda_i\,|v_i\rangle\langle v_i|\,/\mathcal{N}, \tag{23}$$

where $\mathcal{N}$ is a normilization factor to ensure $\mathrm{Tr}(\tilde{\chi}_{\mathcal{S}}^{\rm est}) = 4$. These are what we use in the PAPA construction of the N-qubit gate.

Supplementary Fig. 3 shows an example of the output from our implementation of the PAPA algorithm. For the CNOT gate with single-qubit decoherence described earlier, it plots the difference between the measured (experimentally or in this case by simulation) Choi state, σ_{12}, and the PAPA reconstructed Choi state, ρ_{12}, for the effective process experienced by qubit pair 1-2. The element-wise difference is consistent with the magnitude of the trace distance reported in Supplementary Fig. 2b).

Least-squares minimization requires an initial guess for the χ-matrices, and we choose a decomposition of the ideal three-qubit gate as the initial guess. For the reconstructions presented in the main text, we found that their accuracy was mostly limited by numerical issues, such as the trade-off between the minimization tolerance and computation time. We observed a saturation in the trace distance for solver tolerance below a threshold value of $\epsilon_{\rm tol} = 10^{-7}$, which we attribute to the solver becoming stuck in a local minimum, see Supplementary Fig. 4.

In future work we hope to explore these numerical issues, and implement more efficient and accurate classical algorithms for the PAPA reconstruction. For instance, we would aim to prevent the solver from getting stuck in regions where the gradient of the cost function is below the tolerance threshold, but the solution accuracy is not. One route forward would be to adapt to PAPA more sophisticated optimization algorithms tailored for optimization over positive definite matrices, such as those using gradient descent [4, 5].

Supplementary References

[1] P. V. Klimov, J. Kelly, Z. Chen, M. Neeley, A. Megrant, B. Burkett, R. Barends, K. Arya, B. Chiaro, Y. Chen, A. Dunsworth, A. Fowler, B. Foxen, C. Gidney,

M. Giustina, R. Graff, T. Huang, E. Jeffrey, E. Lucero, J. Y. Mutus, O. Naaman, C. Neill, C. Quintana, P. Roushan, D. Sank, A. Vainsencher, J. Wenner, T. C. White, S. Boixo, R. Babbush, V. N. Smelyanskiy, H. Neven, and J. M. Martinis, Fluctuations of energy-relaxation times in superconducting qubits, Phys. Rev. Lett. **121**, 090502 (2018).

[2] M. Nielsen and I. Chuang, *Quantum Computation and Quantum Information* (Cambridge University Press, Cambridge, UK, 2000).

[3] MATLAB Release 2018a (The MathWorks Inc., Natick, Massachusetts, 2018).

[4] E. Bolduc, G. C. Knee, E. M. Gauger, and J. Leach, Projected gradient descent algorithms for quantum state tomography, npj Quantum Information **3**, 44 (2017).

[5] G. C. Knee, E. Bolduc, J. Leach, and E. M. Gauger, Quantum process tomography via completely positive and trace-preserving projection, Phys. Rev. A **98**, 062336 (2018).
